# Management of a Difficult Case

**Published:** 1872-01

**Authors:** J. C. Cravens


### MANAGEMENT OF A DIFFICULT CASE.

BY J. C. CRAVENS.

I wished to fill the second inferior bicuspid, the cavity being posterior proximal, and so sensitive as to almost throw the patient into spasms. His cheeks were very fleshy, his neck short, and I found it impossible to get a drill in position to sink a retaining point in the cavity, although I had chiseled through from the grinding surface. I determined to fasten entirely upon the grinding surface, but to do this I needed something to consolidate the gold against at the bottom of the cavity. I thought of the solid steel matrix, proposed by somebody in the *Cosmos*, I think, about a year ago. Deeming the solid steel too hard to shape, and believing its unyielding nature rendered it impracticable even after it was shaped, I thought of another simpler appliance. To make *my* matrix I took the annealed end of a thin separating file, which I bent to almost fit the tooth I wished to fill, and placed it in position, and drove a hard hickory wedge tightly between the steel and the first molar. It fitted closely to the margins of the cavity when the pressure of the wedge was brought to bear against it, and was soft enough to well adapt itself and too hard to crowd into the cavity.

I began in the bottom of this flattened cavity and built up my gold until I could anchor it on the grinding surface. I have finished up that filling, and take pleasure in saying that the margins, cervical and all, are as good as I have ever seen in a grinding surface filling of a molar tooth.

